# From Zero to Hero: Type 2 Diabetes Mellitus Patients Hike on the Way of St. James—A Feasibility Study with Analyses of Patients’ Quality of Life, Diabetes Distress and Glucose Profile

**DOI:** 10.3390/ijerph20021417

**Published:** 2023-01-12

**Authors:** Frederike Maria Meuffels, Hans-Peter Kempe, Ulrike Becker, Martin Kornmann, Stephan Kress, Thorsten Kreutz, Christian Brinkmann

**Affiliations:** 1 Department of Preventive and Rehabilitative Sport Medicine, Institute of Cardiovascular Research and Sport Medicine, German Sport University Cologne, 50933 Cologne, Germany; 2 Department of Fitness & Health, IST University of Applied Sciences, 40233 Düsseldorf, Germany; 3 Diabetes Center Ludwigshafen, 67067 Ludwigshafen, Germany; 4 Working Group “Diabetes, Sports and Exercise”, German Diabetes Association (DDG), 10117 Berlin, Germany; 5 Heath & Medical Center, 53123 Bonn, Germany; 6 Center for Diabetes and Hormonal Disorders, 67433 Neustadt, Germany; 7 Vinzentius Hospital, 76829 Landau, Germany

**Keywords:** diabetes, hiking, pilgrimage, quality of life, well-being, diabetes distress, glucose profile, continuous glucose monitoring

## Abstract

This study investigates the feasibility of an accompanied 5-day hiking tour (Way of St. James) for type 2 diabetes mellitus (T2DM) patients and its impact on their quality of life/well-being, diabetes distress and glucose profile. Twenty-three T2DM patients (with and without insulin therapy) participated in the study. The 120 km pilgrimage (from Ferrol to Santiago de Compostela, Spain) was accompanied by three physicians, two diabetes counselors and one sports scientist. Quality of life/well-being was assessed by the World Health Organization’s (WHO)-5 questionnaire, and diabetes distress was evaluated based on the Problem Areas in Diabetes (PAID) scale. The glucose levels of six insulin-treated patients were measured using continuous glucose monitoring (CGM) devices, considering that insulin-treated patients can be at increased risk of exercise-induced hypoglycemia. A significant improvement in quality of life/well-being was reported (*p* < 0.001), while diabetes distress did not change significantly (*p* = 0.203). Only two of the six insulin-treated patients showed moderate hypoglycemic episodes between 0.97% and 5.21% time below range per day, with glucose levels between 53–70 mg/dL. Hiking tours such as the one organized for this study can improve quality of life/well-being without increasing diabetes distress and are considered relatively safe for T2DM patients, even for those being treated with insulin.

## 1. Introduction

Approximately 537 million adults worldwide live with diabetes mellitus. The number of people with type 2 diabetes mellitus (T2DM) is expected to rise even further over the next few years [1]. Diabetes is associated with secondary cardiovascular complications [2,3]. Furthermore, people with diabetes have a higher risk of developing depression [4]. An interaction between diabetes complications at the vascular level and the presence of depression has been demonstrated in a meta-analysis of longitudinal studies [5]. Moreover, due to the current SARS-CoV-19 pandemic, there has been an increase in the number of patients with depression, anxiety and distress [6]. Therefore, measures to counteract the disease and its complications are urgently needed.

A healthy lifestyle can prevent secondary complications that may arise from T2DM or at least delay its progression [7]. Depressive symptoms can be ameliorated by physical activity [8]. Daily physical activity and structured exercise programs are consequently recommended in T2DM therapy guidelines [9]. Unfortunately, adherence to exercise programs among people with T2DM is often very low [10]. 

Jenkins and Jenks [11] assert that hiking is a good solution for improving the adherence of people with T2DM to physical activity programs and describe hiking as a potentially lifelong activity with many health benefits. It is relatively inexpensive and can be easily combined with other activities, is self-paced and is a social activity in nature. Several studies about the positive effects of hiking already exist [12,13]. However, the feasibility of a 5-day hiking pilgrimage tour and its effects on T2DM patients’ glucose profile, quality of life/well-being and diabetes distress have thus far not been explored. 

This study investigates the feasibility of an accompanied hiking tour on the Way of St. James from Ferrol to Santiago de Compostela (Spain) and its psychological and physiological effects on people with T2DM. The pilgrimage route posed a challenge for the participants, with daily hikes of several hours and an approximately 120 km hiking trail in total. 

The following research questions are addressed: What is the effect of a pilgrimage on the quality of life/well-being and diabetes distress in T2DM patients? Are there any health risks associated with the planned hiking tour, especially for insulin-treated patients regarding possible occurrences of exercise-induced hypoglycemia?

## 2. Materials and Methods

### 2.1. Ethics

This preliminary study considers the Declaration of Helsinki and the guidelines of “Good Clinical Practice”. The study was approved by the Ethics Committee of the German Sport University Cologne (Ethics Approval No. 039/2022). All participants were fully informed about the study procedures and gave their written informed consent prior to the commencement of the study. 

### 2.2. Participants

The study was aimed at patients with T2DM (age: >18 years). Participants were recruited via flyers and advertisements on websites. Only those patients who passed a fitness check conducted by a physician were invited to participate in the study. The participants could withdraw their consent to participate in the study at any time without providing a reason. The patients‘ characteristics are presented in Table 1.

Participants shared information about their body mass index (BMI) and glycated hemoglobin (HbA1c) based on their last routine examination at their diabetologist (the routine examination took place 1–2 weeks prior to the commencement of the journey). Most of the patients (n = 20) were taking medication. Of the 10 insulin-treated participants, 5 patients received basal supported oral therapy (BOT) and 5 received basal-bolus insulin therapy (BBT). Detailed information is presented in Table 2. Three physicians, two diabetes counselors and one sports scientist accompanied the pilgrimage.

### 2.3. Study Timeline and Procedures

**Figure 1:**
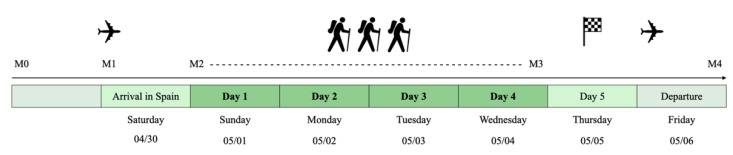
Study design: measurement (M)0, medical examination; M1, questionnaires (World Health Organization (WHO)-5 and Problem Areas in Diabetes (PAID) scale); M2-M3, hiking tour and continuous glucose monitoring (CGM); M4, questionnaires (WHO-5 and PAID).

Figure 1 illustrates the study’s timeline. The participants received training plans to prepare for the hike (see Supplementary Data File S1). The patients confirmed in an interview that they had completed the preparatory program prior to the start of the journey.

Patients were asked to complete two questionnaires (World Health Organization (WHO)-5 and Problem Areas in Diabetes (PAID) scale) via paper and pencil before starting the pilgrimage (shortly before or during the flight from Germany to Spain) as well as after the pilgrimage (during the return flight) to determine their quality of life/well-being and diabetes-related stress. The final score of the WHO-5 ranges between 0 and 100. A score of 0 means very poor well-being and 100 means excellent well-being. PAID scores range from 0 to 100, with 100 representing the highest diabetes-related stress. 

Because strenuous exercise can increase the risk of hypoglycemia in individuals treated with insulin [14], continuous glucose monitoring (CGM) data of insulin-treated patients were analyzed. The participants’ glucose levels were measured during the tour using CGM sensors to determine time below range (TBR). Below-range values were defined as glucose levels <70 mg/dL [15]. At M1, subjects could obtain a CGM device (Freestyle Libre 2, Abbott Diabetes Care Inc., Alameda, USA or DexCom G6 rtCGM, DexCom Inc., San Diego, CA, USA). Of 10 insulin-treated participants, 6 provided glucose data from day 1 to day 4. The other 4 subjects did not provide any data, or their data were incomplete.

CGM data were measured from M2 to M3 (during the 4 days with comparable hiking distances). The participants applied the sensor themselves under medical supervision by a physician or diabetes counselor. Of the 6 included insulin-treated participants, 2 received BOT and 4 BBT.

The following was recommended for insulin-treated patients.

Glucose levels were to be 150–200 mg/dL prior to the hikes. The intake of 1 carbohydrate unit after half an hour of hiking was recommended for patients who had lower values. Patients were to consume 1–3 carbohydrate units per hour in the further course of the physical activity. For patients who received BOT: the basal insulin dose was to be reduced by 20–50%. For patients who received BBT: the morning basal insulin dose was to be reduced by 30–70% for the longer hikes. It was advised to reduce the food bolus by 50–70% during the hike. After the hike, the evening basal insulin dose was to be reduced by 20–50%. The CGM values as well as the trend arrows of individual reactions were continuously monitored and necessary adjustments made. The diabetes team (physicians/diabetes counselors) was always available to provide advice or assistance in case of unusual reactions to the physical activity.

### 2.4. Hiking Route

**Figure 2:**
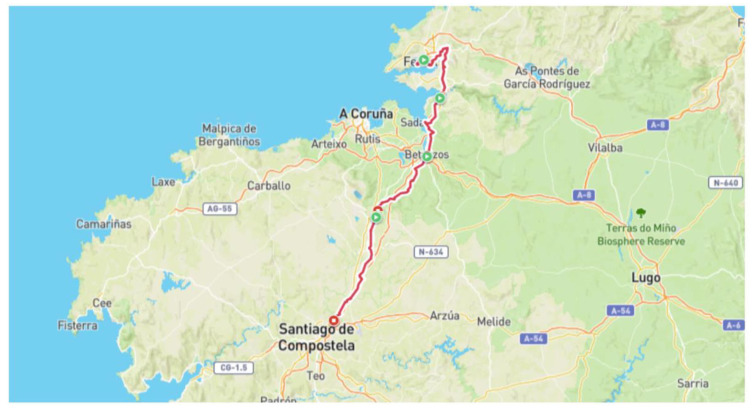
The pilgrim’s path from Ferrol to Formaris and Santiago de Compostela (tracked by the Polar Fitnesswatch).

The patients hiked on the Way of St. James in Spain (Figure 2). The hiking distance on day 1 was about 32 km (from Ferrol to Pontedeume). The total distance on day 2 (between Pontedeume and Betanzos) was around 20 km. On day 3, the distance hiked (between Betanzos and Bruma) was about 25 km. The longest distance (between Bruma and Formaris) was approximately 35 km. On the last day, the hiking distance was about 8 km (from Formaris to Santiago de Compostela). The participants only carried a personal bag. Their luggage was transported to their accommodation by van. 

### 2.5. Data Analysis and Statistical Tests

The Statistical Package for Social Science (IBM SPSS Statistics, version 28, IBM SPSS, Armonk, NY, USA) was used for statistical analyses. To compare the questionnaire results before and after the pilgrimage, the Wilcoxon signed-rank test was used, as data were not normally distributed (indicated by the Shapiro–Wilk test). The significance level was set at *p* < 0.05. CGM data were analyzed descriptively. The CGM sensors measured glucose levels every 15 min. For the 24 h analysis, measurements from 8 AM on the first day until 7.59 AM on the following day were included. Glucose data were exported from the devices/internet clouds to Microsoft Excel (version 2007, Microsoft Corporation, Redmond, WA, USA). 

## 3. Results

All patients arrived at the final destination.

### 3.1. Quality of Life/Well-Being and Diabetes-Related Distress

**Figure 3:**
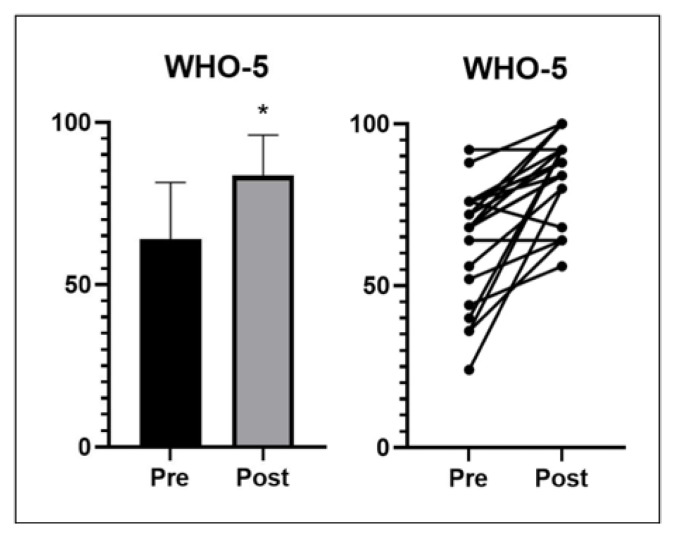
World Health Organization’s (WHO)-5 questionnaire results pre- and post-intervention (n = 23). Means ± standard deviation (SD). * Significantly different from pre-intervention.

**Figure 4:**
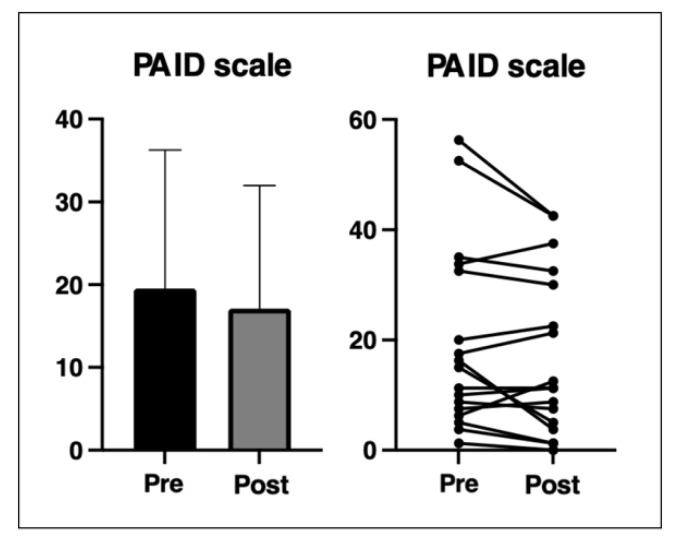
Problem Areas in Diabetes (PAID) scale results pre- and post-intervention (n = 17). Means ± standard deviation (SD).

The pilgrimage increased quality of life/well-being significantly (*p* < 0.001) (Figure 3). No significant difference was detected for the PAID scale results post- vs. pre-pilgrimage (*p* = 0.203) (Figure 4). The PAID scale data of six participants are missing due to unclear or incomplete answers. Although no overall improvement was shown in the PAID scale, significant but very small improvements were seen in the following three subareas (range of each subarea: 0–5): discouragement from the diabetes therapy plan (pre: 0.9 ± 1.1, post: 0.4 ± 0.6; *p* = 0.023), discomfort with social situations related to diabetes care (pre: 0.8 ± 0.8, post: 0.5 ± 0.5; *p* = 0.011) and feeling of being burned out by the constant effort needed to manage diabetes (pre: 0.5 ± 0.8, post: 0.3 ± 0.6; *p* = 0.046). 

### 3.2. Time in Hypoglycemia in Insulin-Treated Participants

**Figure 5:**
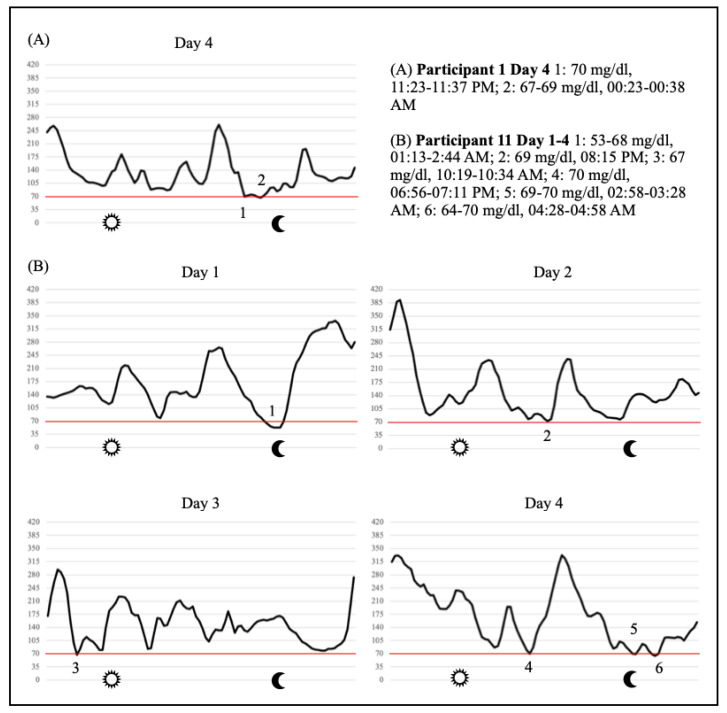
Glucose profiles from two participants with hypoglycemic events.

Only two of six patients showed hypoglycemic episodes. The results are presented in Table 3. The glucose curves of participants No. 1 and No. 11 are illustrated in Figure 5.

## 4. Discussion

This pilot study confirms the feasibility of a 5-day hiking tour with T2DM patients. No adverse events occurred during the tour. Quality of life/well-being increased significantly from pre- to post-intervention. Although such a tour sets higher demands on diabetes management, diabetes distress was not significantly affected. Two of six insulin-treated patients whose glucose values were monitored continuously showed times below range, but without severe hypoglycemic episodes. 

The cutoff point of the WHO-5 for possible clinical depression is 50 [16]. People with diabetes have a higher risk of developing depressive symptoms [4]. Among all participants, five individuals had a score below 50 before the pilgrimage that increased to above 50 in each participant after the pilgrimage, i.e., the patients benefited considerably from the intervention. Many studies have already shown that exercise can positively affect mood and well-being [17,18,19]. However, a systematic review has shown that results about the effects of exercise training on quality of life and mental well-being in people with type 2 diabetes are conflicting [20]. Nevertheless, a negative association between physical activity and depressive symptoms in type 2 diabetes has been reported elsewhere [21]. Lee et al. [22] investigated the effects of physical activities with different intensities. They showed that moderate and vigorous physical activity is better than light physical activity to reduce depression in older adults with diabetes. Furthermore, social support during the pilgrimage may also have played a role. Enggarwati et al. [23] concluded that social support can mitigate depressive symptoms by increasing self-care activities among people with T2DM. In addition, traveling/exercising in a group can have a positive effect on well-being by creating a group feeling and increasing social cohesion [24]. The outdoor experience, i.e., exercising in nature, may also have a more positive effect compared to indoor activities. In this context, Niedermeier et al. [13] found that mountain hiking showed greater effects on activation and fatigue compared to indoor treadmill exercise. 

The average score of the PAID scale was very low, both before and after the trip (the participants’ scores were between 17–19, on average). The cutoff for a potential risk of clinical depression lies at 40 points. Two participants were above this score before and after the pilgrimage, but their score improved substantially through the intervention. Some participants showed higher values after the pilgrimage but were still under the cutoff point. The relatively low overall PAID scores can be rated as positive because physical activity can be stressful, especially for insulin-treated patients with diabetes, given their risk of hypoglycemia and the higher demands set on diabetes self-management to prevent exercise-induced hypoglycemia [25]. According to Finn et al. [26], this may in fact be a reason for non-adherence to physical activity programs or for not following physical activity recommendations. Their concerns about hypoglycemia may have been lower due to the presence of professional medical personnel during the hike. The accompaniment of professional medical personnel and of the other group members might have led to greater subjectively perceived social support. Young et al. [27] assert that diabetes-related stress is lower when greater subjective social support is perceived. The fact that the participants prepared for the hiking tour may be another reason why the PAID scores were relatively low. 

Of the six monitored insulin-treated patients, only two patients (receiving BBT) showed hypoglycemic episodes. Hypoglycemia occurred at irregular times but tended to occur more at night after the long hikes. It should be noted that participant No. 11 experienced regular hypoglycemic events already before the pilgrimage (according to older CGM data, not published). Nevertheless, since hypoglycemia can occur, all participants who are at increased risk of hypoglycemia should wear CGM sensors during such trips to be alerted to low glucose levels early on. 

The present study has some limitations. Due to the pilot nature of the study, a sample size calculation a priori is missing. However, the study results could be useful for the calculation of sample sizes for future studies. Furthermore, a control group is missing. Future studies should be designed as randomized controlled trials. Nevertheless, the study is very innovative and presents a novel approach to the fight against diabetes. Although the long-term effects of regular hiking were not investigated in the present study, it can be assumed that regular hiking can improve low-grade systemic inflammation, triglyceride levels, body weight and insulin sensitivity [12]. Short adventure trips like the one described in the present study may trigger T2DM patients’ interest in hiking and motivate them to continue hiking upon their return home.

## 5. Conclusions

Pilgrimages with T2DM patients can be considered feasible and have beneficial health effects for the participants. Potential risks can be minimized. Accompaniment by physicians and diabetes counselors is recommended. Attention should be paid to controlling glucose levels to prevent (nocturnal) hypoglycemia in insulin-treated patients.

## Figures and Tables

**Table 1:** Participants’ characteristics.

	Total
**Total (n) (%)**	23 (100)
Women (n) (%)	9 (39.1)
Men (n) (%)	14 (60.9)
**Age (years)**	66.3± 6.4
**BMI (kg/m^2^)**	26.2 ± 4.6
**HbA1c (%)**	6.84 ± 0.79
**Diabetes duration (years)**	15.30 ± 14.99

BMI: body mass index, HbA1c: glycated hemoglobin. Means ± standard deviation (SD).

**Table 2:** Participants’ medication.

Blood Glucose Lowering Drugs	Blood Pressure Lowering Drugs	Anti- Hypothyroidism Drugs	Cholesterol Lowering Drugs	Other Drugs/ Supplements
Metformin, dapagliflozin, empagliflozin, semaglutide, sitagliptin, liraglutide, dulaglutide, glibenclamide and insulin (n = 10)	Losartan, amlodipine, valsartan, bisoprolol, candesartan and ramipril	L-thyroxine	Atorvastatin and simvastatin	Gingko special extract EGb761, ASA (acetylsalicylic acid) and tamsulosin
n = 17	n = 9	n = 5	n = 6	n = 5

**Table 3:** Time below range (TBR) during 24 h measurements (08.00 AM–07.59 AM next day) in insulin-treated participants. Participants No.1 and No. 11 showed hypoglycemic episodes.

Participant	Day 1	Day 2	Day 3	Day 4
**1 BBT**	0	0	0	2.08
**5 BOT**	0	0	0	0
**8 BOT**	0	0	0	0
**9 BBT**	0	0	0	0
**11 BBT**	5.21	0.97	1.04	5.14
**19 BBT**	0	0	0	0

## Data Availability

Data are available from the corresponding author on reasonable request.

## Supplementary Materials

The following supporting information can be downloaded at: https://www.mdpi.com/article/10.3390/ijerph20021417/s1, Supplementary Data File S1, Preparatory training program.
