# Supplementary material for: From Zero to Hero: Type 2 Diabetes Mellitus Patients Hike on the Way of St. James—A Feasibility Study with Analyses of Patients’ Quality of Life, Diabetes Distress and Glucose Profile
Source: Int J Environ Res Public Health. 2023 Jan 12;20(2):1417. doi: 10.3390/ijerph20021417 (PMC9861500; doi:10.3390/ijerph20021417)
Supplement: Supplementary file 1 [file ijerph-20-01417-s001.zip › ijerph-2089350-supplementary.pdf]

Supplementary Material File S1.

Training plan

1. Week: 3x 30 min walking
   2x gymnastics: 5 squats, 5 toe stands, 5 knee push-ups, 10 sec forearm plank - 2 rounds

2. Week: 2x 30 min walking, 1x 45 min walking
   2x gymnastics: 7 squats, 7 toe stands, 7 knee push-ups, 15 sec forearm plank- 2 rounds

3. Week: 2x 30 min walking (fast), 1x 60 min walking
   2x gymnastics: 8 squats, 8 toe stands, 8 knee push-ups, 15 sec forearm plank, 5 sec side plank each side - 2 rounds

4. Week: 2x 45 min walking, 1x 90 min hiking
   2x gymnastics: 10 squats, 10 toe stands, 10 knee push-ups, 20 sec forearm plank, 5 sec side plank per side - 2 rounds

5. Week: 1x 60 min walking, 1x 30 min walking (fast), 1x 120 min hiking
   2x gymnastics: 10 squats, 10 toe stands, 10 knee push-ups, 20 sec forearm plank, 10 sec side plank each side - 2 rounds

6. Week: 1x 60 min walking, 1x 45 min walking (fast), 1x 150 min hiking
   2x gymnastics: 10 squats, 10 toe stands, 10 knee push-ups, 25 sec forearm plank, 15 sec side plank each side

7. Week: 2x 60 min walking, 1x 45 min walking (fast)
   2x gymnastics: 15 squats, 15 toe stands, 12 knee push-ups, 30 sec forearm plank, 15 sec side plank each side

8. Week: 1x 60 min walking, 1x 45 hiking (fast), 1x 180 min hiking
   2x gymnastics: 15 squats, 15 toe stands, 12 knee push-ups, 30 sec forearm plank, 15 sec side plank each side

9. Week: 1x 45 min walking, 1x 30 min walking (fast), on 2 consecutive days 120 min and 180 min walking
   2x gymnastics: 20 squats, 15 toe stands, 14 knee push-ups, 35 sec forearm plank, 20 sec side plank each side

10. Week: 1x 60 min walking, 1x 45 min walking (fast), 1x 240 min hiking

2x gymnastics: 20 squats, 15 toe stands, 15 knee push-ups, 35 sec forearm plank, 20 sec side plank each side

11. Week: 1x 90 min walking, 1x 45 min walking (fast),1 x 120 min hiking
2x gymnastics: 20 squats, 15 toe stands, 15 knee push-ups, 40 sec forearm plank, 25 sec side plank each side

12. Week: 1x 90 min walking, 1x 45 min walking (fast), on 2 consecutive days 2x 240 min hiking (at least 1x in the mountains)
2x gymnastics: 20 squats, 20 toe stands, 15 knee push-ups, 45 sec forearm plank, 25 sec side plank each side

13. Week: 1x 60 min walking, 1x 30 min walking (fast), 1x 330 min hiking
2x gymnastics: 20 squats, 20 toe stands, 15 knee push-ups, 50 sec forearm plank, 30 sec side plank each side

14. Week: 1x 30 min walking, 1x 30 min walking (fast), on 2 consecutive days: 2x 240 min hiking (at least 1x in the mountains)
2x gymnastics: 20 squats, 20 toe stands, 15 knee push-ups, 50 sec forearm plank, 30 sec side plank each side

15. Week: 1x30 min walking (fast), 1x 240 min hiking, 1x 360 min hiking
2x gymnastics: 25 squats, 25 toe stand, 15 knee pushups, 55 sec forearm plank, 30 sec side plank each side

16. Week: 2x 60 min walking, 1x 240 min hiking
2x gymnastics: 25 squats, 25 toe stands, 17 knee push-ups, 60 sec forearm plank, 30 sec side plank each side
